# Supplementary material for: Importance of prey size on investigating prey availability of larval fishes
Source: PLoS One. 2021 May 18;16(5):e0251344. doi: 10.1371/journal.pone.0251344 (PMC8130936; doi:10.1371/journal.pone.0251344)
Supplement: S1 Table — (DOCX) [file pone.0251344.s001.docx]

**S1 Table.** Densities of zooplankton, larval fish, and environmental variables of each cruise-station.

| year | month | station | smallsize zooplankton density (ind. m^3^) | copepode density (ind. m^3^) | larval fish density (ind. m^3^) | chlorophlly-a concentration (mg. m^3^) | sea surface temprature (℃) | sea surface salinity (psu) |
| --- | --- | --- | --- | --- | --- | --- | --- | --- |
| 2009 | 7 | 1 | 80308.05 | 162.90 | 6.36 | 2.91 | 25.64 | 33.72 |
| 2009 | 7 | 10 | 9392.56 | 250.18 | 0.22 | 0.20 | 27.28 | 33.46 |
| 2009 | 7 | 11 | 5453.37 | 12.71 | 0.16 | 0.14 | 26.11 | 33.24 |
| 2009 | 7 | 8 | 7586.57 | 36.29 | 0.26 | 1.84 | 23.51 | 33.57 |
| 2010 | 7 | 7 | 44719.22 | 138.32 | 3.46 | 0.51 | 26.07 | 33.72 |
| 2010 | 7 | 10 | 8296.09 | 94.08 | 1.68 | 1.12 | 26.26 | 30.31 |
| 2010 | 7 | 8 | 112137.04 | 2358.95 | 5.66 | 3.47 | 22.13 | 26.80 |
| 2013 | 5 | 1 | 88746.67 | 72.64 | 0.04 | 2.17 | 18.15 | 31.15 |
| 2013 | 5 | 2 | 17125.32 | 495.59 | 0.37 | 1.06 | 21.77 | 34.12 |
| 2013 | 5 | 3 | 30245.30 | 980.06 | 0.24 | 1.09 | 24.41 | 34.14 |
| 2013 | 5 | 4 | 37585.82 | 215.38 | 1.24 | 1.25 | 24.08 | 34.19 |
| 2013 | 5 | 5 | 13611.05 | 1895.17 | 0.24 | 2.05 | 20.55 | 34.50 |
| 2013 | 5 | 6 | 6248.74 | 92.79 | 0.10 | 1.07 | 24.52 | 34.21 |
| 2014 | 5 | 1 | 55016.15 | 451.19 | 1.14 | 4.56 | 20.40 | 30.99 |
| 2014 | 5 | 2 | 31473.11 | 195.22 | 0.59 | 1.63 | 22.72 | 32.71 |
| 2014 | 5 | 3 | 16519.66 | 223.58 | 1.70 | 0.37 | 24.54 | 33.97 |
| 2014 | 5 | 4 | 89440.66 | 289.40 | 1.71 | 0.69 | 26.15 | 33.80 |
| 2014 | 5 | 5 | 18408.15 | 277.66 | 0.24 | 0.79 | 23.51 | 34.26 |
| 2014 | 5 | 6 | 6834.95 | 116.69 | 0.58 | 1.05 | 25.34 | 34.27 |
| 2015 | 7 | 1 | 54788.24 | 400.35 | 0.58 | NA | 25.01 | 32.97 |
| 2015 | 7 | 2 | 10801.41 | 530.09 | 0.01 | NA | 26.46 | 33.98 |
| 2015 | 7 | 3 | 10572.23 | 1231.42 | 0.09 | NA | 27.02 | 33.95 |
| 2015 | 7 | 4 | 29698.26 | 1011.59 | 0.50 | NA | 26.68 | 33.00 |
| 2015 | 7 | 5 | 21108.36 | 473.57 | 0.23 | NA | 25.54 | 34.37 |
| 2015 | 7 | 6 | 7635.02 | 251.50 | 0.05 | NA | 25.33 | 34.38 |
| 2016 | 5 | 1 | 54142.53 | 76.20 | 0.08 | 0.64 | 20.62 | 29.88 |
| 2016 | 5 | 2 | 10522.77 | 250.38 | 0.05 | 2.16 | 22.49 | 32.76 |
| 2016 | 5 | 3 | 49925.31 | 153.31 | 0.08 | 0.71 | 26.45 | 34.62 |
| 2016 | 5 | 4 | 17550.09 | 73.54 | 0.23 | 0.35 | 26.28 | 34.58 |
| 2016 | 5 | 5 | 17712.43 | 580.88 | 0.06 | 0.61 | 24.49 | 34.32 |
| 2016 | 5 | 6 | 2914.36 | 173.06 | 0.04 | 1.38 | 23.13 | 34.52 |
| 2017 | 5 | 1 | 484072.73 | 503.01 | 0.78 | 1.38 | 23.58 | 33.45 |
| 2017 | 5 | 2 | 657341.94 | 383.24 | 1.09 | 0.53 | 23.78 | 34.15 |
| 2017 | 5 | 3 | 176722.58 | 520.64 | 0.98 | 1.01 | 25.28 | 34.44 |
| 2017 | 5 | 4 | 80234.15 | 715.34 | 0.58 | 2.54 | 25.31 | 34.37 |
| 2017 | 5 | 5 | 7708.37 | 697.59 | 0.17 | 1.72 | 20.50 | 34.56 |
| 2017 | 5 | 6 | 57895.38 | 135.42 | 0.15 | 1.34 | 23.36 | 34.45 |
| 2014 | 7 | 7 | 32914.29 | 15.24 | 0.02 | 0.18 | 28.36 | 33.44 |
| 2014 | 7 | 9 | 71666.53 | 954.30 | 0.09 | 11.29 | 24.17 | 30.33 |
| 2014 | 7 | 8 | 45469.56 | 188.37 | 0.15 | 1.33 | 21.28 | 34.11 |
