# Supplementary material for: Importance of prey size on investigating prey availability of larval fishes
Source: PLoS One. 2021 May 18;16(5):e0251344. doi: 10.1371/journal.pone.0251344 (PMC8130936; doi:10.1371/journal.pone.0251344)
Supplement: S2 Table — (DOCX) [file pone.0251344.s002.docx]

**S2 Table.** Size composition of small-size zooplankton of each cruise-station.

| year | month | station | class1 (ind.) | class2 (ind.) | class3 (ind.) | class4 (ind.) | class5 (ind.) | class6 (ind.) |
| --- | --- | --- | --- | --- | --- | --- | --- | --- |
| 2009 | 7 | 1 | 87 | 47 | 11 | 2 | 6 | 2 |
| 2009 | 7 | 10 | 112 | 133 | 80 | 33 | 11 | 7 |
| 2009 | 7 | 11 | 99 | 111 | 50 | 34 | 9 | 4 |
| 2009 | 7 | 8 | 123 | 62 | 20 | 13 | 20 | 7 |
| 2010 | 7 | 7 | 78 | 79 | 43 | 20 | 13 | 12 |
| 2010 | 7 | 10 | 59 | 109 | 62 | 24 | 15 | 6 |
| 2010 | 7 | 8 | 102 | 61 | 15 | 6 | 5 | 1 |
| 2013 | 5 | 1 | 55 | 68 | 20 | 16 | 13 | 7 |
| 2013 | 5 | 2 | 49 | 46 | 22 | 14 | 7 | 2 |
| 2013 | 5 | 3 | 74 | 76 | 27 | 24 | 10 | 8 |
| 2013 | 5 | 4 | 59 | 135 | 93 | 39 | 15 | 10 |
| 2013 | 5 | 5 | 72 | 100 | 51 | 30 | 16 | 8 |
| 2013 | 5 | 6 | 71 | 116 | 50 | 36 | 22 | 14 |
| 2014 | 5 | 1 | 19 | 47 | 32 | 32 | 23 | 15 |
| 2014 | 5 | 2 | 39 | 39 | 29 | 18 | 12 | 2 |
| 2014 | 5 | 3 | 112 | 91 | 38 | 25 | 13 | 10 |
| 2014 | 5 | 4 | 197 | 120 | 46 | 15 | 8 | 6 |
| 2014 | 5 | 5 | 72 | 77 | 46 | 17 | 11 | 16 |
| 2014 | 5 | 6 | 100 | 91 | 76 | 27 | 20 | 8 |
| 2015 | 7 | 1 | 75 | 41 | 11 | 12 | 8 | 4 |
| 2015 | 7 | 2 | 17 | 33 | 36 | 38 | 19 | 8 |
| 2015 | 7 | 3 | 61 | 47 | 19 | 14 | 2 | 3 |
| 2015 | 7 | 4 | 55 | 39 | 15 | 9 | 5 | 0 |
| 2015 | 7 | 5 | 82 | 89 | 34 | 20 | 5 | 2 |
| 2015 | 7 | 6 | 63 | 69 | 34 | 16 | 8 | 2 |
| 2016 | 5 | 1 | 55 | 57 | 24 | 19 | 13 | 2 |
| 2016 | 5 | 2 | 61 | 77 | 30 | 23 | 18 | 14 |
| 2016 | 5 | 3 | 73 | 83 | 45 | 18 | 8 | 6 |
| 2016 | 5 | 4 | 96 | 75 | 31 | 20 | 3 | 7 |
| 2016 | 5 | 5 | 164 | 95 | 39 | 24 | 16 | 4 |
| 2016 | 5 | 6 | 95 | 74 | 36 | 23 | 15 | 10 |
| 2017 | 5 | 1 | 49 | 49 | 26 | 14 | 5 | 1 |
| 2017 | 5 | 2 | 74 | 63 | 35 | 15 | 3 | 2 |
| 2017 | 5 | 3 | 78 | 66 | 26 | 20 | 10 | 7 |
| 2017 | 5 | 4 | 107 | 79 | 42 | 18 | 4 | 2 |
| 2017 | 5 | 5 | 171 | 150 | 59 | 15 | 12 | 3 |
| 2017 | 5 | 6 | 134 | 46 | 25 | 11 | 12 | 7 |
| 2014 | 7 | 7 | 134 | 143 | 58 | 54 | 19 | 3 |
| 2014 | 7 | 9 | 27 | 46 | 30 | 55 | 21 | 6 |
| 2014 | 7 | 8 | 87 | 155 | 67 | 117 | 51 | 20 |
