# Supplementary material for: Importance of prey size on investigating prey availability of larval fishes
Source: PLoS One. 2021 May 18;16(5):e0251344. doi: 10.1371/journal.pone.0251344 (PMC8130936; doi:10.1371/journal.pone.0251344)
Supplement: S3 Table — (DOCX) [file pone.0251344.s003.docx]

**S3 Table.** The results of linear mixed effect models linking larval density with zooplankton density and environmental factors.

| **Model** | **p-value** |
| --- | --- |
| log( larval density ) ~ log( small-size zooplankton ) + (1\|cruise) | 0.001 |
| log( larval density ) ~ log( meso-size zooplankton ) + (1\|cruise) | 0.057 |
| log( larval density ) ~ log( size class 1 ) + (1\|cruise) | 0.00018 |
| log( larval density ) ~ log( size class 2 ) + (1\|cruise) | 0.00055 |
| log( larval density ) ~ log( size class 3 ) + (1\|cruise) | 0.006 |
| log( larval density ) ~ log( size class 4 ) + (1\|cruise) | 0.0507 |
| log( larval density ) ~ log( size class 5 ) + (1\|cruise) | 0.0484 |
| log( larval density ) ~ log( size class 6 ) + (1\|cruise) | 0.368 |
| log( larval density ) ~ sea surface temperature + (1\|cruise) | 0.65 |
| log( larval density ) ~ sea surface salinity + (1\|cruise) | 0.592 |
| log( larval density ) ~ sea surface chlorophyll-a concentration + (1\|cruise) | 0.367 |
