# Supplementary material for: Importance of prey size on investigating prey availability of larval fishes
Source: PLoS One. 2021 May 18;16(5):e0251344. doi: 10.1371/journal.pone.0251344 (PMC8130936; doi:10.1371/journal.pone.0251344)
Supplement: S1 Fig — (DOCX) [file pone.0251344.s004.docx]

**S1 Fig.** Relationships of chlorophyll-a concentration versus log-transformed density of small-size zooplankton (left), mesozooplankton (middle), and larval fish (right). The blue line indicates the significant fitted linear mixed effects model with cruise as a random effect. The shade area indicates confidence intervals.
