# Supplementary material for: Importance of prey size on investigating prey availability of larval fishes
Source: PLoS One. 2021 May 18;16(5):e0251344. doi: 10.1371/journal.pone.0251344 (PMC8130936; doi:10.1371/journal.pone.0251344)
Supplement: Attachment — Submitted filename: PreySize_Response_Letter.docx [file pone.0251344.s005.docx]

**Response Letter**

**Academic editor**

[E1.0] We have got your manuscript reviewed by four different reviewers. Except one, all three reviewers feel that your manuscript has requires major revision. I personally feel that this Ms. is a well-executed work collecting comprehensive data on fish larval abundance and zooplankton and mesoplankton over a period of 8 years. Such field studies provide corroborative evidence for the importance of prey size-larval survival and growth relationship established only in laboratory studies. However, the manuscript needs major revision.

The paper needs thorough investigation for publication. The larval feeding in fish is stage dependent; most fishes do not start external food unless they are ready for it. They do survive with yolk material available on them. The mouth gap of larval fish and prey size are most crucial that determines the relationship between the size fractionation of zooplankton for larval feeding. The present study dealing with relationship between the abundance of fish larvae and size fractionation of zooplankton does not yield enough for publication. The composition of fish larvae as well as their abundance have not been analyzed. This is important as the mouth gap will vary depending upon the species, and therefore a definite relationship could be established. Please take in account of comments given by reviewers and revise the manuscript accordingly.

[E1.0]
We thank the suggestions from the Academic editor. The information of larval fish mouth gape is indeed useful to elucidate the underlying mechanisms of the relationship between larval fish and zooplankton. However, we don’t have such data and this exploration is beyond our scope. In this study, we found a close link between the density of larval fish community and small-size zooplankton. It is likely due to 1) the dominant species of the larval fish community prefers small-size prey, and/or 2) the majority of the sampled larval fish has small body size (hence small mouth gape), which limit the intake of bigger prey. Having the mouth gape data helps to further investigate the reasoning of the observed pattern, but it does not change the strong relationship between larval fish and small-size zooplankton that we reported in this study and does not change the implications of our findings. Nevertheless, it is interesting to further examine how the observed relationship depends on larval fish size. We addressed this as an important future work in the Discussion (*Line 198-205*).

Please see the point-by-point response to reviewers’ comments.

**Reviewer 1**
This is an interesting area of research

[R1.0] The title should be modified.

[R1.0] We thank the reviewer for the suggestion. We chose carefully our current title “Importance of prey size on investigating prey availability of larval fishes” to demonstrate the research gap and the main conclusion of our research. Using empirical data in the Eastern China Sea, we found that the relationship between larval fish and zooplankton density depends on the prey size. Therefore, we feel that this title is appropriate and we would prefer to keep it unless a better title is suggested.

[R1.1] Abstract - line 22, Modify this sentence.

[R1.1] We have modified this sentence to “One arising question is whether the size of zooplankton determines the relationship between zooplankton and larval fish community in natural environments.”

[R1.2] Introduction and Discussion - Recent literature should be consulted. One reference 2017, others are before this period.

[R1.2] Thank you for the suggestion. We added the following literature on the relationship between fish body/mouth size and prey size in the Introduction and Discussion.

*Detmer TM, Einfalt LM, Parkos JJ, Wahl DH. Comparison of mouth morphology and prey size selection among three esocid taxa. Environ Biol Fishes. 2018*

*Egan JP, Gibbs S, Simons AM. Trophic niches through ontogeny in 12 species of Indo-Pacific marine Clupeoidei (herrings, sardines, and anchovies). Mar Biol. 2018*

[R1.3] Materials and Methods - line 62 - The English should be modified. This is also applicable for other places.

[R1.3] We improved the English throughout the manuscript for a better logic flow.

[R1.4] Line 166, it should not be italic.

[R1.4] Revised.

[R1.5] The language requires improvement.

[R1.5] We have improved the language throughout.

This paper may be accepted after minor revision.

Reviewer 2

[R2.1] This essentially a field study is a well-executed work collecting comprehensive data on fish larval abundance and zooplankton and mesoplankton over a period of 8 years. Such field studies provide corroborative evidence for the importance of prey size-larval survival and growth relationship established only in laboratory studies. Authors may please take into account the points I elaborated below while preparing a revision.

[R2.1] We appreciate the reviewer’s positive comments. Please see the point-by-point responses below.

A. Minor errors that need to be corrected:
[R2.2] 1. Line 81: Word given here is 'medium' Shouldn't it be 'median' not medium?

[R2.2] Revised.

[R2.3] 2. Lines 101-103. Size intervals for small zooplankton. The same number cannot be in two different size intervals. (50-75, 75-100). Correct way- Please give size intervals as 50-74, 75-99, 100-124, 125-149, 150-174, 175-200.

[R2.3] The minimum unit of our measurement is lower than 1μm. We therefore added a note at the end of the sentence to specify that the size falls at the upper limit of each class belongs to the next larger group. The sentence reads now: *The six size classes were 50-75 μm, 75-100 μm, 100-125 μm, 125-150 μm, 150-175 μm, and 175-200 μm, respectively (size that falls at the upper limit of each class belongs to the next larger group)* (*Line 101-103*)*.*

[R2.4] 3. Line 124. Not correct to say 'marginally' significant (statistically, something is either significant or it is not). I suggest replacing the sentence with " they were positive but not statistically significant (p~0.057)"

[R2.4] We revised the sentence as below.

Density of copepods exhibited a positive but not statistically significant relationship with larval fish density (*p-value* = 0.057).

[R2.5] 4. Line 145. The statement " All relationships were not significant" might imply that some relationships were significant. To be unambiguous, please state that "None of the relationships were significant".

[R2.5] We revised accordingly.

B. I strongly recommend the following inclusions:

[R2.6] 1. Fig. 2 and 3- For each graph the p value is given. But 'r-square (Coefficient of determination) is a more readily interpretable parameter. I recommend giving r-square values within each graph of Figs. 2 and 3.

[R2.6] Thank you for this suggestion. We added marginal R^2^ values at Fig 2 and Fig 3.

[R2.7] 2. Size frequencies of larval fishes in the samples. Larval fishes were collected using a net of pore size 1000mum. That means, I suppose that the larvae were all larger than 1000um. However, there is no information given about the size frequencies of the fish larvae in collected samples. I recommend that the authors include a figure (as in Fig.1b) showing the size frequencies of larval fish (as they did for small zooplankton in Fig.1b).

[R2.7] We agree that it is interesting to look at size frequencies of larval fish in the samples. However, we don’t have such data and it is beyond our scope to examine how the relationship between larval fish and zooplankton density depends on larval fish size frequencies. Having size frequencies of larval fish data helps to further investigate the reasoning of the observed pattern, but it does not change the strong relationship between larval fish and small-size zooplankton that we reported in this study and does not change the implications of our findings. We addressed this as an important future work in the Discussion (*Line 198-205*).

C. Points or questions that need to be clarified:

[R 2.8] 1. As stated by the authors (and shown as coloured circles in Figs. 2 and 3), data from all the cruises during the sampling period were combined for examining larval abundance-prey food relationships. However, considering that the total sampling period spans ~8 years, the reader might wish to have some idea about the "inter-cruise" variation in fish larval abundance and, if possible, in small and mesozooplankton abundance also in the sampled areas. At the appropriate place in the text, authors could provide intercruise variation as a 'coefficient of variation' (C.V.).

[R 2.8] Thank you for the suggestion. The inter-cruise variations for small-zooplankton, mesozooplankton, and larval fish are 1.43, 0.58, and 0.92, respectively. We have added this information in the Methods (*Line 98-100*).

[R 2.9] 2. Relationship between mesozooplankton (copepods?) and larval abundance. It was positive but not statistically significant. But p value is 0.057, so close to significance level of 0.05). Does this suggest that the largest size class of the fish larvae might be feeding preferentially on mesozoolankton? Also, I note that the largest small zooplankton size classes (sizes 5 and 6, Fig.1b) formed less than 10% of zooplankton abundance.

[R 2.9] We speculate that larger size classes of larval fish may be feeding on mesozooplankton. However, as we do not have the data of larval fish size frequency, we are not able to draw this conclusion. Regarding the size frequencies of small-size zooplankton, size 5 and 6 form a small portion of the zooplankton abundance, but it is not related to the size-specific relationships we observed. For example, there is a significant relationship between larval fish and size class 5 (*p* = 0.048), whereas there is no significant relationship between larval fish and size class 6 (*p* = 0.368).

[R 2.10] 3. Relationship with chlorophyll. I assume that the dominant food of small zooplankton included green algae and diatoms, whose densities are generally reflected in chlorophyll concentration in the water. Therefore, I would have expected indirectly positive correlations among chlorophyll- zooplankton, and larval fish abundance. Authors may add a few lines giving probable explanation.

[R2.10] We ran additional analyses and found that chlorophyll-a concentration has a positive relationship with the density of mesozooplankton. In contrast, such relationship is lacking with small-size zooplankton (see below graphs). It is likely because small-size zooplankton consume not only phytoplankton but also protists. Thus, the sources of food could come through not only grazing food chain but also microbial loop, which could not be fully captured by chlorophyll-a concentration. Importantly, mesozooplankton density does not have a significant relationship with larval fish density (Figure 2). These results may explain why we did not detect a relationship between chlorophyll-a concentration and larval fish density.

**Figure S1** Relationships of chlorophyll-a concentration versus log-transformed density of small-size zooplankton (left), mesozooplankton (middle), and larval fish (right). The blue line indicates the significant fitted linear mixed effects model with cruise as a random effect. The shade area indicates confidence intervals.

We added the graphs as supplementary Figure S1 and revised the Discussion as below.

*The lack of relationship between chlorophyll-a concentration and larval fish density suggests that chlorophyll-a concentration does not indicate food availability of larval fish in our study area. Interestingly, chlorophyll-a concentration has a positive relationship with mesozooplankton density, but exhibits a none-significant relationship with small-size zooplankton (****Fig S1****). It is likely because small-size zooplankton consume not only phytoplankton but also protists. Thus, the sources of food could come through not only grazing food chain but also microbial loop, which could not be fully captured by chlorophyll-a concentrations. Importantly, mesozooplankton density does not have a significant relationship with larval fish density (****Fig 2****), explaining the lack of relationship between chlorophyll-a concentration and larval fish density (Line 189-197).*

D. Information or data on prey sizes consumed by larval fish

[2.11] The authors rightly pointed out in the Discussion part (Lines 195-200) the importance of knowing the sizes of zooplankton consumed by the larvae for understanding the mechanism underlying the observed relationship between larval abundance and zooplankton densities. I wish to know... is it possible that the authors could conduct a gross gut content analysis of a sample of larval fish that they had collected and preserved? Even gape size measurements of preserved larvae may not be too difficult or time-consuming. These two pieces of data, if collected and included in the paper, will strengthen the arguments that they cited in Discussion. I strongly urge the authors to explore the feasibility of accomplishing the additional work that I suggested.

[2.11] Thank you for the suggestion. The information on gut content or mouth gape of fish samples is useful to estimate the size ratio between larval fish and its prey, and prey selectivity of larval fish. However, these objectives are beyond the scopes of our study. The strong relationship between the density of larval fish and small-size zooplankton is independent of the information on larval fish mouth gape and gut content. Nevertheless, it is interesting to further examine how the observed relationship depends on larval fish size. We highlighted this point as an important future work in the discussion (*Line 213-220*).

Reviewer 3

[R3.1] The research paper is coherently created. However inadvertent grammatical errors have to be rectified. For example:
In addition, the positive relationship between larval fish density and the density of
120 small-size zooplankton were observed in most cruises, whereas the relationships with copepods were
121 inconsistent among cruises; some cruises showed hump-shaped (i.e. May 2013) or even negative
122 relationships (i.e. July 2016).
"The positive relationship" has to be followed with "Was" and not "were"

[R3.1] We revised these grammatical errors accordingly throughout the manuscript.
